# Comparison of surgical outcomes between primary and secondary brain abscess

**DOI:** 10.3892/mi.2024.160

**Published:** 2024-05-09

**Authors:** George Fotakopoulos, Charalampos Gatos, Konstantinos Paterakis, Vasiliki Epameinondas Georgakopoulou, Theodosis Spiliotopoulos, Grigorios Christodoulidis, Pagona Sklapani, Nikolaos Trakas, Adamantios Kalogeras, Kostas N. Fountas

**Affiliations:** 1 Department of Neurosurgery, General University Hospital of Larissa, 41221 Larissa, Greece; 2 Department of Pathophysiology, National and Kapodistrian University of Athens, 11527 Athens, Greece; 3 Department of General Surgery, University Hospital of Larissa, Larissa 41110, Greece; 4 Department of Biochemistry, Sismanogleio Hospital, 15126 Athens, Greece

**Keywords:** brain abscess, primary brain abscess, secondary abscess, surgical outcome

## Abstract

Brain abscess (BA) constitutes 1-8% of intra-cerebral tumors, and thus the present study aimed to compare the surgical outcomes of patients with primary and secondary BA. The present retrospective study examined 32 of cases BA who underwent surgery in a local institution between February, 2013 and December, 2023. All patients received intravenous antibiotic therapy according to the antibiogram for antimicrobial susceptibility. In total, 32 patients were separated into two groups as follows: Group A (16 patients, 50%) with primary abscess and group B (16 patients, 50%) with secondary abscess. Of the 32 patients included in the study, 23 (71.8%) were males, and the median age was 55.3 years. On the whole, the present study demonstrates that a multidisciplinary approach involving a combination of often multiple surgical procedures and prolonged antibiotic medication may improve the functional outcome if the underlying pathology allows for a functional outcome.

## Introduction

Brain abscess (BA) constitutes 1-8% of all intracerebral tumors (1). Even though there has been immense progress made in diagnostic and treatment techniques, BA remains one of the reasons for unfavorable outcomes (2,3). However, modern neurosurgical techniques, such as the use of neuronavigation in brain surgical procedures, as well as non-invasive, accurate radiological techniques, such as magnetic resonance spectroscopy, and more targeted antibiotic therapy have succeeded in leading to notable improvements in the surgical outcomes of patients with BA (1,4).

Primary or hematogenous extended abscesses more frequently have an odontogenic or cardiogenic origin or are observed in immunocompromised patients and are usually located at the edge of gray and white matter (5). Over the past 15 years, the frequency of primary BAs has decreased, while the frequency of post-traumatic or post-operative (secondary) BAs has increased (6).

However, to the best of our knowledge, there is currently no data available concerning the suitable therapeutic management for BA, probably due to the low incidence. Moreover, to the very best of our knowledge, no consensus or randomized therapeutic trials have been published comparing the surgical outcomes of patients with primary and secondary BA (7).

The present study thus aimed to address this gap in the literature by comparing the surgical outcomes of patients with primary and secondary BA, in order to determine the factors related to unfavorable outcomes.

## Patients and methods

### Patients and data collection

The present retrospective study included 32 patients with BA who underwent surgery in a local institution (University Hospital of Larissa, Larissa, Greece) between February, 2013 and December, 2023. The Institutional Review Board (IRB) of the University of Thessaly, Greece, the School of Medicine, and the School of Health Sciences approved its IRB Number, 2697/15-01-2024, finalized by the 38th General Assembly on January 28, 2023. The present study was in line with the Declaration of Helsinki (1995; as revised in Edinburgh 2000). Written informed was obtained from all the included patients or their next-of-kin or the parents/legal guardians prior to the surgery.

These patients were separated into two groups as follows: Group A (primary abscess) and group B (secondary or iatrogenic-related abscess). Additional classifications in group B were made following the neurosurgical intervention: Traumatic brain injury (TBI), tumors, edema and intracranial hematoma (ICH).

Data collection was carried out on the basis of the following inclusion criteria: Patients aged >8 years that underwent a surgical procedure for primary or secondary BA (following neurosurgical interventions: TBI, tumors, edema and ICH) detection. The exclusion criteria consisted of clinical and laboratory signs of severely reduced liver and kidney function, immunosuppressive patients, impaired gastrointestinal absorption, pregnancy, or the suspicion of resistant causative bacterial strains. The participants were categorized based on the following clinical or radiographical data retrieved from the medical archives when available: Sex, age, Glasgow Coma Scale (GCS) upon admission, diabetes, hypertension, alcohol use, drug abuse, history of surgical procedures [external ventricular drain (EVD) and the site of craniotomy/decompressive craniectomy (DC) in one site or bilateral], and the timing of intravenous (IV) antibiotic treatment prior to neurosurgical intervention (Table I). The clinical and radiographic data were collected according to the clinical presentation (headache, neurological deficit, or fever) and brain imaging (computed tomography or magnetic resonance imaging), which was consistent with a BA, according to the hospital radiologist.

All patients received IV antibiotic therapy according to the antibiogram for antimicrobial susceptibility. The follow-up ranged from 1 to 10 years, commencing on the day of hospital discharge, and was the same in both groups.

The primary outcome was the GCS score at 1 month following neurosurgical intervention, and the secondary outcomes were fever duration, hospital stay, intensive care unit stay, mortality and cure (Table II).

### Statistical analysis

The Statistical Package for the Social Sciences ver. 11 (SPSS, Inc.) was used for all statistical analyses. The assessment of the normality of the allocation of variables was carried out using the Shapiro-Wilk test, while Fisher's exact test was performed for the categorical variables. The Mann-Whitney U test was used for the evaluation of continuous data. Multivariate linear regression analysis was performed in order to examine the effects of multiple variables on the dependent variable. It was possible to use receiver operating characteristic (ROC) analysis to ascertain the utility of surgery for the primary vs. the secondary BA as a predictor of adverse outcomes (mortality). A P-value <0.05 was considered to indicate a statistically significant difference.

## Results

In total, 32 patients who underwent surgical procedures for BAs with primary or secondary detection were registered in the present study. These patients were separated into two groups as follows: Group A (16 patients, 50%) with a primary abscess and group B (16 patients, 50%) with a secondary abscess. Of the 32 included patients, 23 (71.8%) were males, and the median age was 55.3 years (range, 9-82 years). The baseline demographic characteristics of the study participants are presented in Table I. The microorganisms isolated from the tissue biopsied during surgery are presented in Table III.

Univariate analysis revealed that there was a statistically significant difference between the two groups with respect to the following parameters: EVD and bilateral craniotomy/DC; the timing of IV treatment initiation prior to the intervention; and fever duration (P<0.05, Table IV).

**Figure 1:**
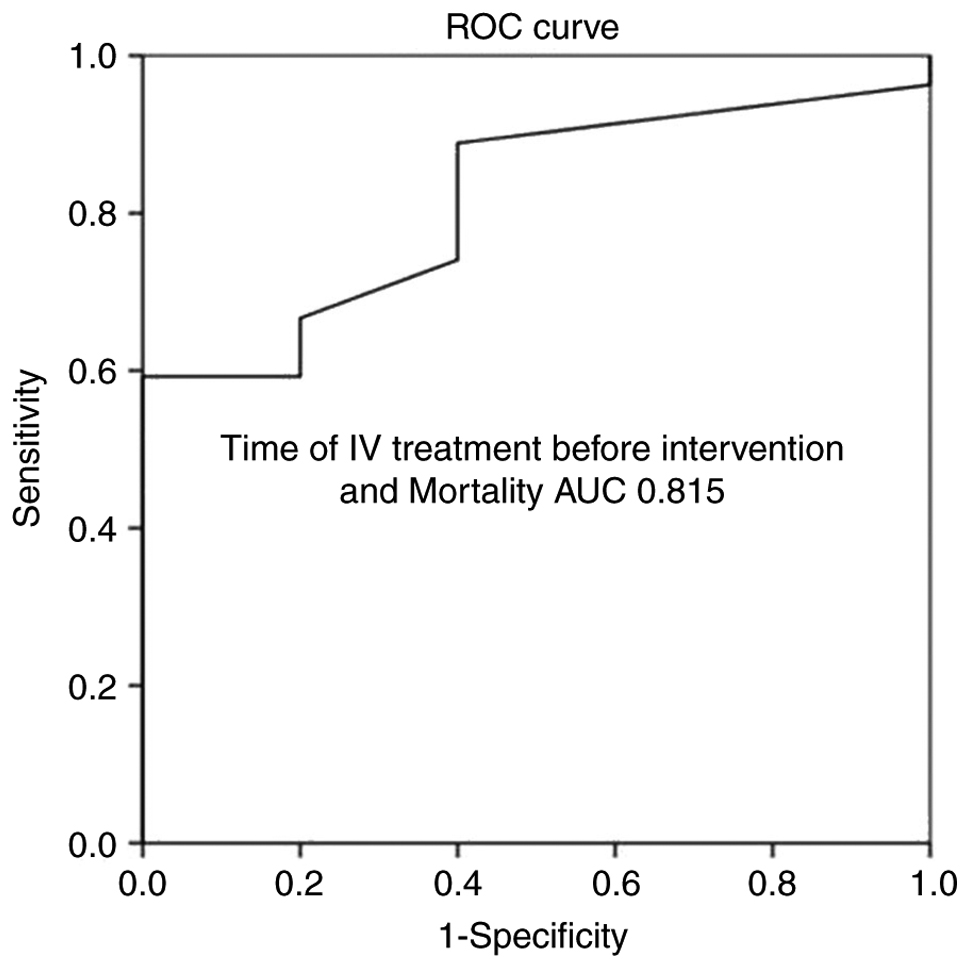
ROC analysis for the time of IV treatment prior to intervention and mortality, predicting that an interval of 21 days inception from the brain abscess surgical evacuation was related with favorable outcome. AUC, 0.815. AUC, area under the curve; ROC, receiver operative characteristic; IV, intravenous.

**Figure 2:**
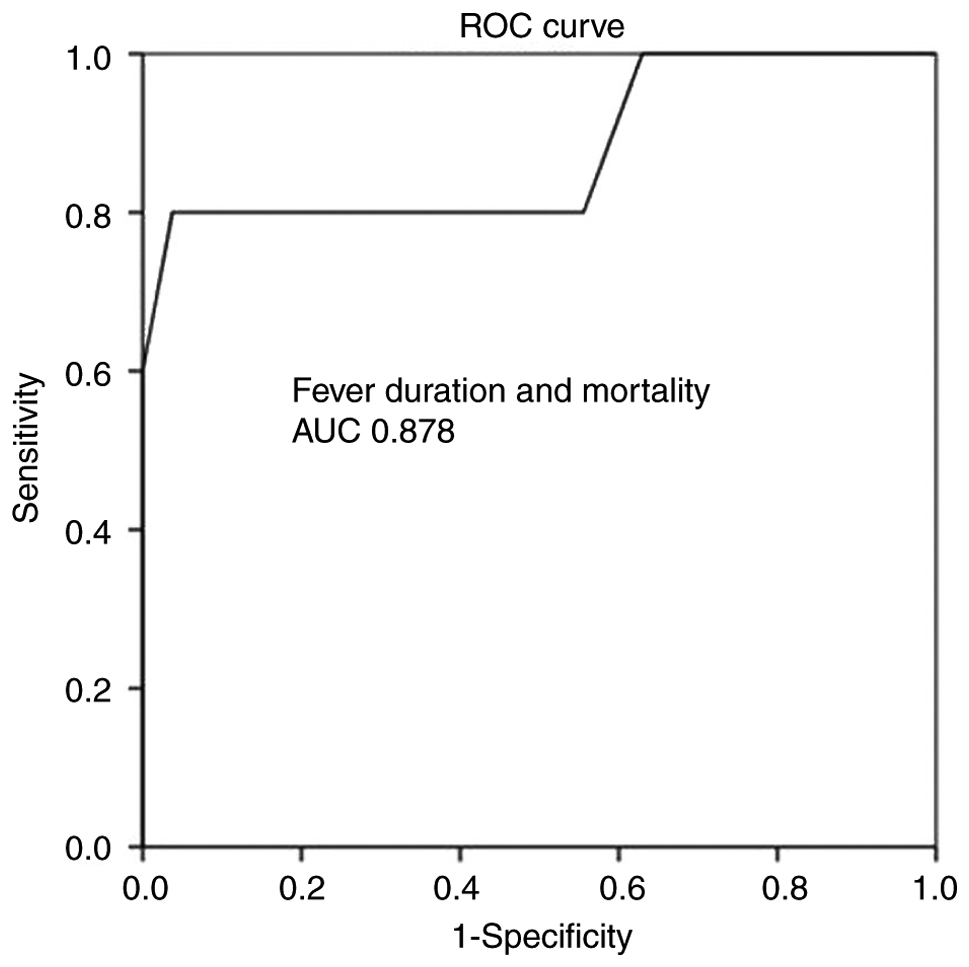
ROC analysis for fever duration and mortality, predicting that an interval of 2.5 days duration following the brain abscess surgical evacuation was related with an unfavorable outcome. AUC, 0.878. AUC, area under the curve; ROC, receiver operative characteristic.

Multivariate linear regression analysis (Table V) disclosed that all aforementioned parameters were associated with mortality. More specifically, ROC analysis for IV treatment initiation prior to intervention demonstrated that an interval of 21 days prior to the surgical procedure had better dispersion (97% sensitivity and 100% specificity) as evaluated by an area under the curve (AUC) standard error (SE) of 0.815 (0.085) and P<0.05, as shown in Table VI and Fig. 1. In addition, ROC analysis for fever duration revealed that an interval of 2.5 days following the surgical intervention had a better dispersion (100% sensitivity and 93% specificity), as indicated by an AUC (SE) of 0.878 (0.109) and P<0.05 (Table VI and Fig. 2).

## Discussion

In the present study, patients who underwent surgical procedures for BA with secondary detection had a more favorable outcome compared with those that presented with primary BA. In other words, patients who underwent surgery for a BA, EVD treatment, or a bilateral craniotomy had worse outcomes. In addition, the timing of IV treatment prior to the intervention and fever duration were independent outcome predictors. Notably, patients had a better outcome if the IV treatment commenced 21 days prior to the surgical intervention. Moreover, if the fever persisted for 2.5 days post-operatively, this was a good prognostic factor for patient outcomes.

BA constitutes a highly controversial and challenging entity, even in sophisticated healthcare facilities with ample resources, and is associated with high mortality and morbidity rates (2,3). A multidisciplinary approach is mandatory, as is long-term follow-up, particularly in environments with limited resources where the BA incidence is higher (8). However, although in low- and middle-income countries (LMICs) BA accounts for 8% of all intercranial masses and this figure declines to only 2% for higher-middle-income countries (HMICs), primary BA in the child population is very common in LMICs, while in HMICs only sporadic cases have been reported (9). Another major difference between LMICs and HMICs is the surgical procedure, as in the former settings, the majority of cases are treated via burr hole aspiration (9).

Major procedures, such as craniotomy or craniectomy have been reported in a number of studies (10-13). In a previous cohort study, only one patient was treated via burr hole aspiration (10). According to the current guidelines, antimicrobials should be withheld until aspiration or the excision of BA in patients without severe disease if neurosurgery can be carried out within 24 h following the radiological diagnosis (10). In the patient sample in the present study, however, the surgical removal of the BA was usually preceded by antibiotic therapy, as the majority of the patients were referred to the clinic following an ENT (ear, nose and throat) consultation and had been receiving antimicrobial medication for days or weeks. Hakan *et al* (14) reported a significant association between a high fever (>38.5˚C) and mortality.

According to the available literature, a poor prognosis of patients with BA is more likely in immunocompromised patients, as well as in those with a history of diabetes and a low GCS score (7). In general, patients presenting with a lower GCS score have a poorer prognosis and often develop fatal complications (1,3). In the present study, there was no association between diabetes and GCS at the time of admission.

Despite the surgical management of BA, antibiotics are very efficient in both the early and late periods (1,3). The limited efficacy of antibiotics, mainly after the incapsulated stage, is due to the failure to achieve the sufficient therapeutic absorption of the antibiotic into the abscess cavity (1,3). On the other hand, the implications of long-term antibiotic use, such as increased liver and kidney burden, intestinal microbiome disorders, side-effects and effects on the immune system, constitute major concerns, particularly in patients with BA (1,3). In addition, antibiotic resistance, which is a global issue, enhances the susceptibility to secondary infections and has negative effects on the microbiota and health (1,3). Similar to all these reports, in the present study, management with antibiotics was one of the main parameters associated with favorable outcomes. More precisely, it was found that IV antibiotic administration 21 days prior to the surgical removal of the BA was related to improved outcomes.

In extant literature, fever is the most commonly reported symptom of BA (15). In the present study, a fever duration exceeding 2.5 days post-operatively was related to a poor response to treatment and thus with an unfavorable outcome.

The present study had several limitations that should be noted when interpreting the reported findings. The main limitation stems from its retrospective nature, which increased the risk of possible errors in collecting and interpreting the data from the clinical history, such as seizures or steroid administration. In addition, another limitation was that all patients were treated with IV antibiotics and there was no control group.

In conclusion, currently, BA is still considered a highly complex pathological entity with different pathophysiological aspects to be considered to treat their catastrophic sequelae. A multidisciplinary approach involving a combination of often multiple surgical procedures and a prolonged antibiotic administration may improve the functional outcomes of patients if the underlying pathology does not preclude this possibility. The present study revealed that patients who underwent surgical procedures for BA with secondary detection had a more favorable outcome compared with those with primary BA and a history of EVD treatment and/or bilateral craniotomies. In addition, it was found that the timing of IV treatment initiation prior to the intervention and fever duration could predict the outcomes of patients. Thus, patients had a better outcome if the IV treatment commenced 21 days prior to the surgical intervention. Moreover, if the fever persisted for not >2.5 days following the surgery, this was a good prognostic factor for patient outcomes.

Aspects that are still controversial and should be assessed further through high-quality studies include the following: The surgical approach for major craniotomies or DCs with full excision of BA or minimal image-guided stereotactic aspiration, the duration of IV antibiotic medication (6-8 weeks), when to switch to oral antibiotics, and for how long.

## Figures and Tables

**Table I:** Baseline demographic characteristics of the patients in the present study.

Parameters	All patients, n=32 (100%)	Group A, n=16 (50%)	Group B, n=16 (50%)	P-value
Age, mean ± SD (years)	55.3±16.7	54.6±16	55.9±17	0.720
Sex (male), n (%)	23 (71.8)	12 (37.5)	11 (34.3)	0.694
GCS score upon admission, mean ± SD	11.4±2.5	11.3±2.7	11.5±2.4	0.741
Diabetes mellitus, n (%)	4 (12.5)	3 (9.3)	1 (3.1)	0.285
Hypertension, n (%)	11 (34.3)	6 (18.7)	5 (15.6)	0.710
Alcohol use, n (%)	5 (15.6)	2 (6.2)	3 (9.3)	0.626
Drugs abuse, n (%)	1 (3.1)	0 (0)	1 (3.1)	0.310
History of surgical procedure				
EVD	1 (3.1)	0 (0)	1 (3.1)	0.310
Site of craniotomy/DC				**<0.05**
One site, n (%)	14 (43.7)	0 (0)	14 (43.7)	0.283
Bilateral, n (%)	2 (6.2)	0 (0)	2 (6.2)	0.144
Time of IV treatment before intervention, mean ± SD (days)	10.0±6.6	15.9±3.9	4.1±1.3	**<0.05**

Values in bold font indicate statistically significant difference (P<0.05). EVD, external ventricular drain; GCS, Glasgow Coma Scale; SD, standard deviation; DC, decompressive craniectomy; IV, intravenous.

**Table II:** Outcomes of the patients following treatment.

Parameter	All patients, n=32 (100%)	Group A, n=16 (50%)	Group B, n=16 (50%)	P-value
GCS after neurosurgical intervention, mean ± SD	12.4±3.5	12.0±3.8	12.7±3.2	0.595
Fever duration, mean ± SD (days)	9.3±9.4	9.2±6.2	9.3±11.9	**<0.05**
Hospital stay, mean ± SD (days)	58.2±8.25	63.4±3.8	53.0±8.2	**<0.05**
ICU stay, mean ± SD (days)	13.1±13.2	14.2±14.5	12.0±12.2	0.661
Mortality, n (%)	5 (15.6)	0 (0)	5 (15.6)	**<0.05**
Cured, n (%)	27 (84.3)	16(50)	11 (34.3)	**<0.05**

Values in bold font indicate statistically significant difference (P<0.05). GCS, Glasgow Coma Scale; SD, standard deviation; ICU, intensive care unit.

**Table III:** Isolated microorganisms in biopsy of the patients operated on for abscess.

Microorganism	Group A, n=16	Group B, n=16
*Acinetobacter baumannii*, n(%)	1 (3.1)	5 (15.6)
*Enterococcus*, n (%)	1 (3.1)	2 (6.2)
*Candida auris*, n (%)	1 (3.1)	3 (9.3)
*Bacteroides fragilis*	2 (6.2)	0 (0)
*Klebsiella pneumoniae*, n (%)	3 (9.3)	4 (12.5)
*Streptococcus intermedius*, n (%)	1 (4.3)	6 (18.7)
*Streptococcus constellatus*, n (%)	2 (6.2)	0 (0)
*Propionibacterium*, n (%)	5 (15.6)	4 (12.5)
*Serratia*, n (%)	1 (3.1)	3 (9.3)
*Staphylococcus aureus*, n (%)	-	6 (18.7)

Polymicrobial cases: 2 cases with *Acinetobacter baumannii* and *Klebsiella pneumoniae*; 2 cases with *Acinetobacter baumannii* and *Enterococcus*; 2 case with *Staphylococcus aureus* and *Serratia*; 1 case with *Bacteroides fragilis* and *Klebsiella pneumoniae*; and 2 cases with *Candida auris* and *Klebsiella pneumoniae* and *Streptococcus intermedius.*

**Table IV:** Univariate analysis for mortality.

Parameters	Survivors n=27	Non-survivors, n=5	P-value
Age, mean ± SD (years)	55.5±17.3	59.2±13.3	0.604
Sex (male), n (%)	1 (3.1)	4 (12.5)	0.660
GCS score of admission, mean ± SD	11.4±2.7	11.2±1.0	0.249
Diabetes mellitus, n (%)	3 (9.3)	1 (3.1)	0.581
Hypertension, n (%)	10 (31.2)	1 (3.1)	0.461
Alcohol use, n (%)	5 (15.6)	0 (0)	0.295
Drugs abuse, n (%)	1 (3.1)	0 (0)	0.662
History of surgical procedure			
EVD	0 (0)	1 (3.1)	**<0.05**
Site of craniotomy/DC			
One site, n (%)	11 (34.3)	3 (9.3)	0.425
Bilateral, n (%)	0 (0)	2 (6.2)	**<0.05**
Time of IV treatment prior to intervention, mean ± SD (days)	11.1±6.6	4.2±1.7	**<0.05**
GCS after neurosurgical intervention, mean ± SD	12.4±3.7	12.4±2.5	0.748
Fever duration, mean ± SD (days)	6.5±5.8	24.2±11.6	**<0.05**
Hospital stay, mean ± SD (days)	57.9±7.7	59.8±11.7	0.959
ICU stay, mean ± SD (days)	11.6±12.9	21.2±13.8	0.189

Values in bold font indicate statistically significant difference (P<0.05). GCS, Glasgow Coma Scale; EVD, external ventricular drain; DC, decompressive craniectomy; SD, standard deviation; ICU, intensive care unit.

**Table V:** Multivariate linear regression analysis for mortality.

			95% CI for Exp(B)	
Parameter	P-value	Exp(B)	Lower	Upper
Surgical procedure				
EVD	**<0.05**	-0.300	-1.108	-0.143
Site of craniotomy/DC				
Bilateral, n (%)	**<0.05**	0.603	0.569	1.239
Time of IV treatment prior to the intervention, mean ± SD (days)	**<0.05**	-0.312	-0.026	-0.008
Fever duration, mean ± SD (days)	**<0.05**	0.701	0.021	0.031

Values in bold font indicate statistically significant difference (P<0.05). SD, standard deviation; CI, conﬁdence interval; DC, decompressive craniectomy; EVD, external ventricular drain.

**Table VI:** ROC analysis for absorption.

Parameters	Area	Std. error	95% CI, lower-upper	P-value
Surgical procedure				
EVD	0.600	0.155	0.297-0.90	0.484
Site of craniotomy/DC				
Bilateral, n (%)	0.700	0.154	0.399-1.000	0.161
Time of IV treatment prior to intervention, mean ± SD (days)	0.815	0.085	0.648-0.981	**<0.05**
Fever duration, mean ± SD (days)	0.878	0.109	0.665-1.000	**<0.05**

Values in bold font indicate statistically significant difference (P<0.05). SD, standard deviation; CI, conﬁdence interval; DC, decompressive craniectomy; EVD, external ventricular drain.

## Data Availability

The datasets used and/or analyzed during the current study are available from the corresponding author on reasonable request.
